# NP-59 SPECT/CT Imaging in Stage 1 Hypertensive and Atypical Primary Aldosteronism: A 5-Year Retrospective Analysis of Clinicolaboratory and Imaging Features

**DOI:** 10.1155/2013/317934

**Published:** 2013-10-21

**Authors:** Yi-Chun Chen, Jainn-Shiun Chiu, Yuh-Feng Wang

**Affiliations:** ^1^Division of Nephrology, Department of Internal Medicine, Dalin Tzu Chi Hospital, Buddhist Tzu Chi Medical Foundation, No. 2 Minsheng Road, Dalin Township, Chiayi County 622, Taiwan; ^2^School of Medicine, Tzu Chi University, Hualien 97004, Taiwan; ^3^Department of Nuclear Medicine, Saint Paul's Hospital, Taoyuan City 330, Taiwan; ^4^Department of Nuclear Medicine, Dalin Tzu Chi Hospital, Buddhist Tzu Chi Medical Foundation, Chiayi 622, Taiwan

## Abstract

*Objective.* We retrospectively analyzed all primary aldosteronism (PA) patients undergoing NP-59 SPECT/CT imaging with regard to their clinicolaboratory and imaging features, investigation, and outcomes. *Material and Methods.* 11 PA patients who presented to our hospital for NP-59 SPECT/CT imaging between April 2007 and March 2012 and managed here were analyzed. *Results.* Among 11 PA patients, eight (73%) had stage 1 hypertension, three (27%) stage 2 hypertension, four (36%) normal plasma aldosterone concentration, nine (82%) nonsuppressed plasma renin activity (PRA), six (55%) normal aldosterone-renin-ratio (ARR), eight (73%) serum potassium ≧3 mEq/L, seven (64%) subclinical presentation, seven (64%) negative confirmatory testing, and four (36%) inconclusive results on CT scan and seven (64%) on planar NP-59 scan. All 11 (100%) patients had positive results on NP-59 SPECT/CT scan. Two (18%) met typical triad and nine (82%) atypical triad. Among nine atypical PA patients, three (33%) had clinical presentation, six (67%) subclinical presentation, six (67%) negative confirmatory testing, and four (44%) inconclusive results on CT scan and six (67%) on planar NP-59 scan. All patients had improved outcomes. Significant differences between typical and atypical PA existed in PRA and ARR. *Conclusions.* NP-59 SPECT/CT may provide diagnostic potential in stage 1 hypertensive and atypical PA.

## 1. Introduction 

Primary aldosteronism (PA) is the most common surgically curable form of secondary hypertension and has also been documented to trigger harmful cardiovascular events independent of hypertension [1]. PA classically presents with typical triad of elevated plasma aldosterone concentration (PAC), suppressed plasma renin activity (PRA), and high aldosterone-renin-ratio (ARR). Saline infusion or captopril tests are used to confirm the diagnosis. It has been shown that PA reaches at least 11.2% prevalence among newly diagnosed hypertensive patients [2], when determinations of ARR, PAC, and PRA are used as screening tools. 

Screening for PA is generally recommended in subjects with drug-resistant hypertension or stage 2 hypertension [3] according to JNC 7 [4], despite hypokalemia or normokalemia, because of a high frequency (~21%) of PA in stage 2 essential hypertension [5]. However, recent data have shown that PA is not uncommon in normotensive, prehypertensive, and stage 1 hypertensive patients [6–9]. Patients with normotensive and subclinical PA may represent as an early, milder form of PA which may subsequently develop into hypertension on followup and lead to more aldosterone-dependent cardiovascular morbidity than essential hypertension [7, 8, 10]. Emerging evidence also has shown that PA patients may vary widely in their clinicolaboratory features [5], including atypical triad of normal PAC [6, 11–14], nonsuppressed PRA [6, 11], and normal ARR [6, 11]. In addition, significant numbers of prehypertensive PA may have a subclinical picture [8, 9]. Moreover, low-renin hypertension and PA may also share elevated ARR [11, 13]. Thus, detection of the early or milder form of PA represents a particular challenge for clinicians.

PA lateralization determines the treatment strategy. Several tools for PA lateralization are currently in use. However, adrenal computed tomography (CT) or magnetic resonance imaging (MRI) findings alone are insufficient for lateralization [15], since subtle hyperfunctioning nodules or hyperplasia may exist in normal-appearing adrenal glands [16]. Although adrenal venous sampling (AVS) is the gold standard for lateralization, successful sampling remains technically demanding. Moreover, potential complications may arise including hemorrhage and excessive radiation exposure [17], and inconclusive or discordant results between CT/MRI and AVS may exist [18]. 

^131^I-6*β*-iodomethyl norcholesterol (NP-59) has high affinity for adrenocortical tissue; however, planar scanning is limited by poor resolution. Single-photon emission computed tomography/computed tomography (SPECT/CT) is a significant technical innovation that employs an integrated dual-head gamma camera and a low-dose, noncontrast, nondiagnostic CT scanner. Since SPECT/CT integrates the simultaneous functional and anatomic evaluation of adrenal dysfunction, it has been proven superior over planar scanning in adrenal gland scintigraphy and allows clinicians to identify small adrenal lesions [19, 20]. In a recent retrospective study including 27 patients with clinically confirmed PA [18], NP-59 SPECT/CT has been shown to reach a high sensitivity (up to 81.8%) and diagnostic accuracy and is considered the primary lateralization tool when CT and AVS results are inconclusive. However, only two case reports have addressed the role of NP-59 SPECT/CT in atypical PA [16, 21] and stage 1 hypertensive PA [16], and the limited results appear to be promising. 

Dalin Tzu Chi General Hospital is the first hospital possessing NP-59 SPECT/CT modality in the Yunlin-Chiayi-Tainan area and integrating this imaging in evaluation of PA patients. In the present study, we aimed to retrospectively analyze all PA patients presenting to our hospital between April 2007 and March 2012 and undergoing NP-59 SPECT/CT imaging in terms of their clinicolaboratory and imaging features, investigation, and outcomes.

## 2. Material and Methods

### 2.1. Setting

Dalin Tzu Chi General Hospital is a regional teaching hospital with 948 beds serving an extensive population in Chiyi County, Yunlin County, and Tainan County.

### 2.2. Study Design and Patients

All patients who presented to our hospital and underwent NP-59 SPECT/CT imaging and were pathologically confirmed as PA between April 2007 and March 2012 were retrospectively evaluated with regard to their clinicolaboratory and imaging features, interventions, and outcomes. Demographic data (age and sex) and clinical information were reviewed from the medical notes and analyzed.

### 2.3. Definitions

The severity of hypertension was staged according to JNC 7 criteria [4] with stage 1 equivalent to 140 to 159 mm Hg systolic over 90 to 99 mm Hg diastolic and stage 2 as >160/100 mm Hg. Clinical PA was defined by stage 1 or 2 hypertension with serum potassium less than 3 mEq/L or stage 2 hypertension with serum potassium greater than 3 mEq/L. Subclinical PA was defined by stage 1 hypertension with serum potassium greater than 3 mEq/L or normokalemia (serum potassium > 3.5 mEq/L). PAC and PRA were measured by radioimmunoassay using commercially available kits (Diasorin Inc., MN, USA). Normal ranges for PAC and PRA were 3.7–24 ng/dL and 0.15–2.33 ng/mL/h, respectively. An ARR >30 was considered elevated [17]. All drugs that might affect the ARR were discontinued 2 weeks before performing confirmatory testing. Confirmatory testing included an IV saline load (2 L of 0.9% NaCl infused over 4 h), which was considered positive if posttest PAC was greater than 10 ng/dL [3]. Alternatively, a captopril test (25 mg of captopril) was performed and considered positive if posttest PAC suppression after 2 hours was greater than 30% [3]. Kaliuria was defined by transtubular potassium concentration gradient (TTKG) > 4. Typical PA was defined as PA patients who met the triad of elevated PAC, suppressed PRA, and high ARR. Atypical PA was defined as PA patients who had normal PAC, or nonsuppressed PRA, or normal ARR.

### 2.4. NP-59 Planar and SPECT/CT Imaging

A dexamethasone suppression regimen (1 mg orally four times daily) was initiated seven days prior to tracer injection and was continued throughout the imaging procedure and for five days postinjection [22]. In order to block thyroid uptake of free I-131, subjects were also given five drops daily of Lugol's solution three days before the start of imaging and daily until the end of the imaging period. All drugs that might interfere with NP-59 uptake were discontinued for four weeks prior to imaging [22]. NP-59 scanning was performed on days 1 through 5 to obtain planar images after intravenous injection of 1.5 mCi (56 MBq) of NP-59. SPECT/CT scanning was performed on days 2 through 5 with a dual-head gamma camera and a low-dose nondiagnostic CT (Infinia Hawkeye 4, GE Healthcare, Milwaukee, WI, USA) to obtain merged SPECT/CT images. This low-dose nondiagnostic CT operates at 140 mv–2.5 mA.

### 2.5. Imaging and Pathological Interpretation

The NP-59 planar and SPECT/CT images were interpreted after a consensus reading by two board-certified nuclear medicine physicians who were unaware of the clinical data. Aldosteronism on the affected side(s) was diagnosed if there was early visualization of the tracer before the fifth postinjection day and if intense uptake (greater than that seen in the liver) was noted on the image [2]. Adrenal CT imaging with 3 mm thin cuts was interpreted by a board-certified radiologist unaware of clinical data. Ten patients underwent laparoscopic adrenalectomy and one was treated with spironolactone. Histopathological examinations were performed by a board-certified pathologist unaware of clinical data.

### 2.6. Outcome Evaluation

All patients were followed up for at least six months following adrenalectomy or medical treatment. Improvement was defined as well-controlled blood pressure (BP) without antihypertensive medications or a decrease in the dose or class of antihypertensive medications, and/or normalization or decrease of PAC, PRA, and serum potassium levels.

### 2.7. Statistical Analysis

Categorical data are expressed as number (percentage) and continuous data as median (range). The difference between typical and atypical PA patients (Table 3) was compared using the Mann-Whitney *U* test for continuous variables. A two-sided *P* value less than 0.05 was considered statistically significant. All data were analyzed using SPSS version 13.0 (SPSS Inc., Chicago, IL). This study was approved by our Institutional Review Board. Written informed consent was obtained from all subjects. 

## 3. Results 

A total of 11 PA patients (6 men and 5 women, median age: 55 years; range: 27–75 years) using NP-59 SPECT/CT imaging were shown in detail in Table 1. Eight patients had adrenal adenoma, one adrenal micronodule, one focal nodular hyperplasia, and one bilateral adrenal hyperplasia without surgery.

### 3.1. Analysis according to Hypertension Stage

Among 11 PA patients (Table 2), eight (73%) had stage 1 hypertension, three (27%) stage 2 hypertension, four (36%) normal PAC, nine (82%) nonsuppressed PRA, six (55%) normal ARR, eight (73%) serum potassium ≧3 mEq/L, seven (64%) subclinical presentation, seven (64%) negative confirmatory testing, and four (36%) inconclusive results on CT scan and seven (64%) on planar NP-59 scan. All 11 (100%) patients had positive results on NP-59 SPECT/CT scan. Stage 1 hypertensive PA patients had a higher percentage of normal PAC, nonsuppressed PRA, normal ARR, serum potassium ≧3 mEq/L, subclinical presentation, negative confirmatory testing, and negative results on CT scan.

### 3.2. Analysis according to Typical versus Atypical Triad

Integrated and quantitative analyses of all PA cases according to typical versus atypical triad can be gained from Tables 3 and 4. Among 11 PA patients, two (18%) had typical triad and nine (82%) atypical triad. Among atypical PA patients, three (33%) had clinical presentation, six (67%) subclinical presentation, six (67%) negative confirmatory testing, and four (44%) inconclusive results on CT scan and six (67%) on planar NP-59 scan. All atypical PA patients had positive results on NP-59 SPECT/CT scan and improved outcomes. Benefit of NP-59 SPECT/CT could be summarized as one point, that is, the disclosure of adrenal lesions in typical or atypical PA with clinical or subclinical presentation despite negative confirmatory testing and/or inconclusive results on traditional lateralization modalities. Among 11 PA patients using NP-59 SPECT/CT imaging, median systolic BP was 150 mm Hg, median diastolic BP 90 mm Hg, median PAC 26.8 ng/dL, median PRA 1.31 ng/mL/h, median ARR 18, and median serum potassium 3.4 mEq/L. There were significant differences in PRA and ARR between typical and atypical PA.

### 3.3. Outcome Followup

**Figure 1:**
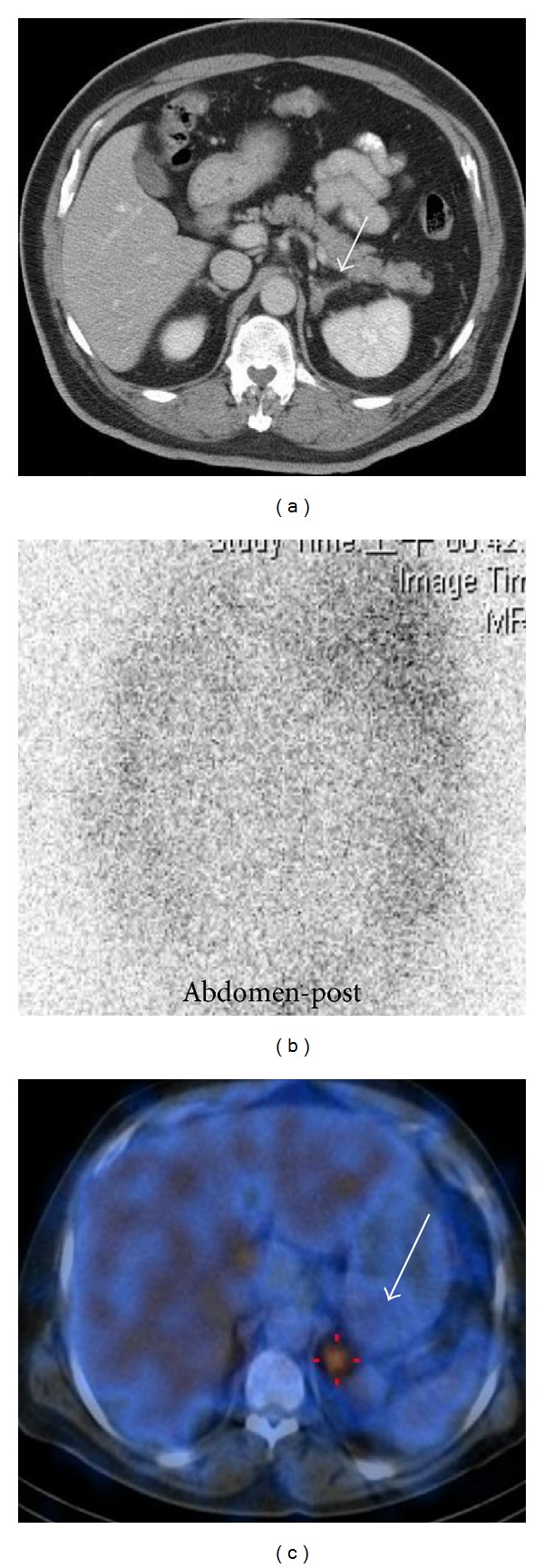
A 56-year-old male patient (case 4) with stage 1 hypertension for 3 years presents with atypical triad of PA and normokalemia. Contrast adrenal CT (b) shows a 1.2 cm nodular lesion in left adrenal gland (arrow). NP-59 96 h planar posterior imaging (b) indicates no radiotracer uptake within either adrenal gland, but fused SPECT/CT (c) indicate intense uptake in the left adrenal (arrow), consistent with left adrenal aldosteronism. His hypertension cured following left adrenalectomy.

On followup (Table 1), eight stage 1 hypertensive PA patients were cured of their hypertension following treatment and three stage 2 hypertensive PA patients had improvement in hypertension. It is worth noting that patient 4 (Figure 1) shared a clinical presentation similar to essential hypertension, which made it difficult to access the subject for PA but was ultimately diagnosed with PA by a positive NP-59 SPECT/CT result.

## 4. Discussion

A methodology to detect atypical PA and stage 1 hypertensive PA using NP-59 SPECT/CT imaging against general screening for typical PA has been presented. This strength of this approach lies in its higher sensitivity and diagnostic accuracy, as well as its safety with no contrast exposure and very little radiation exposure from the nondiagnostic CT scanner. Our preliminary results indicated three clinical benefits of NP-59 SPECT/CT in PA. The first is to discover stage 1 hypertensive PA despite the presence of atypical triad or/and negative confirmatory testing. The second is to confirm the diagnosis of atypical PA when there is clinical suspicion. The third is to detect invisible adrenal lesions not found by conventional imaging.

In the present study, PA patients using NP-59 SPECT/CT imaging were featured as stage 1 hypertension, atypical triad, subclinical presentation, serum potassium ≧ 3 mEq/L (normokalemia, 46%), negative confirmatory testing, and inconclusive results on CT and planar NP-59 scanning (Tables 1 and 3). It seems reasonable to expect that clinical presentation and typical triad predominate in stage 2 hypertensive PA. However, a significant proportion of stage 1 hypertensive PA was accompanied with subclinical and atypical PA and seemed to be less easy access because of the obstacle to negative confirmatory testing, inconclusive results on CT and planar NP-59 scanning (Table 2). These findings were consistent with the prevailing concept that most PA patients exhibit an attenuated form of the disease and normokalemia, and only a minority exhibit typical triad and hypokalemia [5, 7]. This could lead to marked underdiagnosis of PA. Emerging circumstantial evidence has also supported the notion of neurohormonal heterogeneity and progression over time in PA until the “autonomous” nature of aldosterone secretion results in hypertension [12, 13] and that PA should be considered as a continuum of pathological disorders [5]. 

Given that normokalemic or mildly hypertensive PA may have low positive yield on confirmatory testing [23, 24], this would explain why up to 63% of stage 1 hypertensive PA patients in this study had negative confirmatory testing. Given that adrenal CT or planar NP-59 findings alone are insufficient for lateralization due to their low accuracy in detecting subtle hyperfunctioning nodules or hyperplasia [15], this would explain a significant proportion of inconclusive results on these traditional modalities in stage 1 hypertensive PA in this study. Given that normotensive PA may reflect an early or milder form of PA [7, 9], NP-59 SPECT/CT appears feasible for the diagnosis of stage 1 hypertensive PA, which was not documented in the literature.

Next, we analyzed the differences between typical and atypical PA (Tables 3 and 4). Subclinical presentation, stage 1 hypertension, and negative confirmatory testing seemed to predominate in atypical PA. If traditional imaging fails to support the clinical suspicion of PA, NP-59 SPECT/CT seems to provide diagnostic potential for atypical PA. In addition, significant differences between typical and atypical PA existed in PRA and ARR.

Stage 1 hypertensive PA and atypical PA seem to be not uncommon. The number of stage 1 hypertensive and atypical PA patients increased from four and five, respectively, in 2007–2010 [24] to eight and nine, respectively, till March 2012 in our hospital. Given the higher prevalence of PA among prehypertensive and stage 1 hypertensive patients [8, 9], NP-59 SPECT/CT appears to provide significant improvement in diagnosis. It remains unclear, however, whether it is cost effective to screen for normotensive and mildly hypertensive PA using NP-59 SPECT/CT. Given that modest adrenal hormonal autonomy, as exhibited in clinically silent normokalemic PA, is associated with significant morbidity [25] and that hyperaldosteronism is fairly common in hypertension [14] and is associated with aldosterone-dependent cardiovascular morbidity, long-term care with antihypertensives, and cardiovascular complications, increased efforts to identify such cases appear justified [26, 27]. In this study, eight stage 1 hypertensive patients were cured of their hypertension.

In the SPECT/CT systems currently commercially available, we adopted the GE Hawkeye hybrid system with a low-dose nondiagnostic CT scan that is a low cost option [28] and aids the diagnosis and therapeutic planning in various clinical situations [18, 19]. The radiation exposure from this 2.5 mA CT scan of an abdomen nondiagnostic localization is small (about 0.5 mSv) compared with the dose received from the use of spiral CT [29]. Therefore, SPECT/CT may be suited to play a major role in noninvasive and safe characterization of subtle adrenal lesions.

This study had some limitations. First, this was a retrospective analysis. Second, AVS was not available for all patients. Despite its usefulness, successful sampling of both adrenal veins remains technically demanding and potentially harmful and thus has been limited largely to major tertiary centers. Despite these limitations, our findings are clinically significant. It is increasingly being recognized that PA is not confined to stage 2 hypertensive patients but also common in stage 1 or mildly hypertensive patients and that atypical PA is common. This evidence poses a challenge for the clinicians to the existed guideline that screening for PA should be recommended to stage 2 hypertensive patients. Noninvasive NP-59 SPECT/CT appears to have promising potential in identifying stage 1 hypertensive and atypical PA. 

## 5. Conclusion

In conclusion, this study demonstrates diagnostic potential of noninvasive NP-59 SPECT/CT in the diagnosis of stage 1 hypertensive and atypical PA. A prospective scale-up study is warranted to validate our findings in the future.

## Figures and Tables

**Table 1:** Detailed profile of all PA cases using NP-59 SPECT/CT imaging between April 2007 and March 2012 (*n* = 11).

Case	Age (year)	Sex	BP (mm Hg)	Class of anti-hypertensives	HTN Stage*	K^#^ (mEq/L)	PAC^#^ (ng/dL)	PRA^#^ (ng/mL/hr)	ARR	TTKG	Confirmatory testing	CT (site, mm)	NP-59		Pathology (mm)	Improved outcomes
													Planar	SPECT/CT		
1	55	F	140/90	1	1	3.24	31.9	2.52	13	8.8	Saline loading (N) Captopril (N)	Normal	N	R	Micronodule (0.8)	PAC, K, BP
2	48	F	145/80	2	1	4.01	26.8	0.06	447	ND	Saline loading (N) Captopril (N)	L (17)	L	L	Adenoma (17)	PAC, PRA, BP
3^‡^	57	M	170/100	4	2	2.79	37.2	0.32	116	6.2	Saline loading (N)	L (puffy, 9)	N	L	Focal nodular Hyperplasia	PAC, PRA, K, BP
4	56	M	144/90	1	1	4.14	25.3	1.31	12	ND	ND	L (12)	N	L	Adenoma (10)	PAC, BP
5	39	M	206/115	4	2	2.2	27.5	1.68	16	8.2	ND	R (14)	N	R	Adenoma (12)	PAC, K, BP
6^‡^	27	F	150/88	2	1	4.32	29.3	1.62	18	ND	Captopril (N)	Normal	Faint	Bil	No operation^†^	PAC, BP
7	53	M	145/63	2	1	2.95	37.7	0.02	1885	ND	Saline loading (P) Captopril (P)	L (20)	N	L	Adenoma (18)	PAC, K, BP
8	61	F	150/93	1	1	3.84	19.9	0.39	51	ND	Captopril (N)	L (29)	L	L	Adenoma (26)	BP
9	63	M	136/79	1	1	3.4	17.4	1.39	13	ND	Saline loading (N) Captopril (N)	L (21)	L	L	Adenoma (22)	K, BP
10	40	F	150/90	1	1	3.1	5.36	1.99	2.7	6.3	ND	R (20)	R	R	Adenoma (20)	BP, K
11	75	M	181/92	6	2	3.9	8.42	0.21	39	ND	Captopril (N)	L (puffy, 10)	N	L	Adenoma (10)	BP

Abbreviations: BP: blood pressure; HTN: hypertension; K: potassium; PAC: plasma aldosterone concentration; PRA: plasma renin activity; ARR: aldosterone-renin-ratio; TTKG: transtubular potassium gradient; F: female; M: male; ND: not done; P: positive; N: negative; L: left; R: right; Bil: bilateral.

^#^Normalrange of PAC, PRA, and serum K is 3.7–24 ng/dL, 0.15–2.33 ng/mL/h, and 3.5 to 5.0 mEq/L, respectively.

*HTN stage according to JNC 7 report.

^†^Only spironolactone therapy.

^‡^Patient 3 had stage 3 chronic kidney disease and patient 6 had stage 4 chronic kidney disease.

**Table 2:** Qualitative analysis by HTN stage (*n* = 11).

Characteristics	All (*n* = 11)	Stage 1 HTN (*n* = 8)	Stage 2 HTN (*n* = 3)
Class of antihypertensives			
≧3	3 (27)	0 (0)	3 (100)
<3	8 (73)	8 (100)	0 (0)
PAC			
Elevated	7 (64)	5 (63)	2 (67)
Normal	4 (36)	3 (37)	1 (33)
PRA			
Suppressed	2 (18)	2 (25)	0 (0)
Nonsuppressed	9 (82)	6 (75)	3 (100)
ARR			
Elevated	5 (45)	3 (37)	2 (67)
Normal	6 (55)	5 (63)	1 (33)
Serum K (mEq/L)			
Normal (>3.5)	5 (46)	4 (50)	1 (33)
3 ≦ Serum K < 3.5	3 (27)	3 (38)	0 (0)
2 ≦ Serum K < 3	3 (27)	1 (12)	2 (67)
Presentations			
Clinical	4 (36)		
Stage 2 HTN + 2 ≦ Serum K < 3		—	2 (cases 14, 16)
Stage 2 HTN + Serum K > 3.5		—	1 (case 22)
Stage 1 HTN + 2 ≦ Serum K < 3		1 (case 18)	—
Subclinical	7 (64)		
Stage 1 HTN + 3 ≦ Serum K < 3.5		3 (cases 12, 20, 21)	—
Stage 1 HTN + Serum K > 3.5		4 (cases 13, 15, 17, 19)	—
Confirmatory testing			
Positive	1 (9)	1 (12)	0 (0)
Negative	7 (64)	5 (63)	2 (67)
Not done	3 (27)	2 (25)	1 (33)
CT lesion			
Positive (nodule)	7 (64)	6 (75)	1 (33)
Adrenal puffiness	2 (18)	0 (0)	2 (67)
Negative	2 (18)	2 (25)	0 (0)
NP-59 Planar			
Positive	4 (36)	4 (50)	0 (0)
Faint	1 (9)	1 (12)	0 (0)
Negative	6 (55)	3 (38)	3 (100)
NP-59 SPECT/CT			
Positive	11 (100)	8 (100)	3 (100)

Abbreviations are the same as Table 1. Data are expressed as number (percentage).

**Table 3:** Quantitative analysis between typical and atypical PA cases (*n* = 11).

Variable	All (*n* = 11)	Typical (*n* = 2)	Atypical (*n* = 9)	P*
SBP (mm Hg)	150 (135–206)	145 (145)	150 (136–206)	0.58
DBP (mm Hg)	90 (63–115)	72 (63–80)	90 (63–115)	0.07
PAC (ng/dL)	26.8 (5.36–37.7)	32.2 (26.8–37.7)	25.3 (5.36–37.2)	0.33
PRA (ng/mL/h)	1.31 (0.02–2.52)	0.04 (0.02–0.06)	1.39 (0.21–2.52)	0.036
ARR	18 (2.7–1885)	1165 (447–1885)	16 (2.7–116)	0.036
Serum K (mEq/L)	3.4 (2.2–4.32)	3.4 (2.95–4.01)	3.4 (2.2–4.32)	1.00

Abbreviations: SBP: systolic blood pressure; DBP: diastolic blood pressure. Other abbreviations are the same as Table 1. Data are expressed as median (range).

*P < 0.05as significant.

**Table 4:** Integrated analysis by triad.

Triad				Confirmatory Testing^1^	Case				NP-59				Improved outcome
		Presentation					CT			Planar		SPECT/CT	
		Serum K	HTN stage			P^2^	Puffy	N	P	Faint	N	P	
Typical (*n* = 2)													
PAC↑, PRA↓, ARR↑	Clinical	<3	1	P	Case 7	*✓*					*✓*	*✓*	*✓*
	Subclinical	>3.5	1	N	Case 2	*✓*			*✓*			*✓*	*✓*

Atypical (*n* = 9)													
PAC↑, PRA−, ARR↑	Clinical	<3	2	N	Case 3 (Kaliuria)		*✓*				*✓*	*✓*	*✓*
PAC↑, PRA−, ARR−	Clinical	<3	2	ND	Case 5 (Kaliuria)	*✓*					*✓*	*✓*	*✓*
	Subclinical	3–3.5	1	N	Case 1 (Kaliuria)			*✓*			*✓*	*✓*	*✓*
		>3.5	1	N	Case 6			*✓*		*✓*		*✓*	*✓*
				ND	Case 4	*✓*					*✓*	*✓*	*✓*
PAC−, PRA−, ARR↑	Clinical	>3.5	2	N	Case 11		*✓*				*✓*	*✓*	*✓*
	Subclinical	>3.5	1	N	Case 8	*✓*			*✓*			*✓*	*✓*
PAC−, PRA−, ARR−	Subclinical	3–3.5	1	N	Case 9	*✓*			*✓*			*✓*	*✓*
				ND	Case 10 (Kaliuria)	*✓*			*✓*			*✓*	*✓*

Abbreviations are the same as Table 1.

^1^Indicates either saline loading or captopril testing.

^2^Indicates adrenal nodule.
